# Determining the effectiveness of High Resolution Melting analysis for SNP genotyping and mutation scanning at the *TP53 *locus

**DOI:** 10.1186/1471-2156-10-5

**Published:** 2009-02-17

**Authors:** Sonia Garritano, Federica Gemignani, Catherine Voegele, Tú Nguyen-Dumont, Florence Le Calvez-Kelm, Deepika De Silva, Fabienne Lesueur, Stefano Landi, Sean V Tavtigian

**Affiliations:** 1 Department of Biology – Genetics via Derna 1, 56126 University of Pisa, Pisa, Italy; 2 Genetic Susceptibility group, International Agency for Research on Cancer, Lyon, France; 3 Idaho Technology Inc, Salt Lake City, Utah, USA

## Abstract

**Background:**

Together single nucleotide substitutions and small insertion/deletion variants are the most common form of sequence variation in the human gene pool.

High-resolution SNP profile and/or haplotype analyses enable the identification of modest-risk susceptibility genes to common diseases, genes that may modulate responses to pharmaceutical agents, and SNPs that can affect either their expression or function. In addition, sensitive techniques for germline or somatic mutation detection are important tools for characterizing sequence variations in genes responsible for tumor predisposition. Cost-effective methods are highly desirable. Many of the recently developed high-throughput technologies are geared toward industrial scale genetic studies and arguably do not provide useful solutions for small laboratory investigator-initiated projects. Recently, the use of new fluorescent dyes allowed the high-resolution analysis of DNA melting curves (HRM).

**Results:**

Here, we compared the capacity of HRM, applicable to both genotyping and mutation scanning, to detect genetic variations in the tumor suppressor gene *TP53 *with that of mutation screening by full resequencing. We also assessed the performance of a variety of available HRM-based genotyping assays by genotyping 30 *TP53 *SNPs. We describe a series of solutions to handle the difficulties that may arise in large-scale application of HRM to mutation screening and genotyping at the *TP53 *locus. In particular, we developed specific HRM assays that render possible genotyping of 2 or more, sometimes closely spaced, polymorphisms within the same amplicon. We also show that simultaneous genotyping of 2 SNPs from 2 different amplicons using a multiplex PCR reaction is feasible; the data can be analyzed in a single HRM run, potentially improving the efficiency of HRM genotyping workflows.

**Conclusion:**

The HRM technique showed high sensitivity and specificity (1.0, and 0.8, respectively, for amplicons of <400 bp) for mutation screening and provided useful genotyping assays as assessed by comparing the results with those obtained with Sanger sequencing. Thus, HRM is particularly suitable for either performing mutation scanning of a large number of samples, even in the situation where the amplicon(s) of interest harbor a common variant that may disturb the analysis, or in a context where gathering common SNP genotypes is of interest.

## Background

Together Single Nucleotide Polymorphisms (SNPs), rare single nucleotide substitutions, and small insertion/deletion mutations constitute the most common forms of sequence variation in the human genome. For example, Nickerson *et al*. [1] have estimated that the density of common SNPs (with a frequency greater than 1%) is about 1 per 300 bp in the overall human gene pool. Furthermore, deep resequencing studies have demonstrated that the number of rare single nucleotide substitutions and small insertion/deletion variants vastly outnumber common SNPs [2,3].

During the last decade, SNPs have essentially replaced microsatellites for linkage and/or association studies [4,5] and genome-wide association studies with phase 2 and phase 3 confirmations have now provided overwhelming evidence of association on common SNPs with a number of diseases [6,7]. SNPs are also becoming of interest in pharmacogenetics, because some of them are associated with significant differences in biological response to pharmaceutical agents [8,9].

Heavy interest in SNPs has led to the development of different genotyping methods: some of them are targeted to the analysis of one or few SNPs [10,11], and others are designed to scan the whole genome [12,13]. Modern genotyping equipment has driven the per genotype cost for very large-scale SNP genotyping studies quite low. In addition, clonal sequencing technologies may drive the cost of moderate sensitivity resequencing studies very low [14,15]. However, these technologies are actually geared to what are essentially industrial scale genetic studies and arguably to not provide useful solutions for small laboratory investigator-initiated projects.

Interest in fast and reliable methods of mutation screening is increasing as well. Such methods are desirable for case-control mutation screening studies and high-throughput somatic (tumor) mutation screening studies [16,17], aiding the identification of new genes involved in carcinogenesis. They are also desirable for detecting genes responsible for drug-resistance in micro-organisms [18], and for detecting genes that modify growth, resistance to parasites, or yield in plants [19].

Many techniques have been developed to discover genomic variation, including those based on HPLC (High Performance Liquid Chromatography), electrophoretic conformational changes, and enzymatic or chemical cleavage reactions [20]. The goal of these screening techniques is to reduce the use of DNA sequencing and control costs while maintaining sensitivity and specificity. The HRM technique has been used to mutation scan the coding sequences of several clinically important genes [21-26]. For instance, 3 studies have reported mutation screening of *TP53 *exonic regions [21,22,27]. In this manuscript, we describe lessons learned from a larger scale application of HRM to mutation screening and genotyping at the entire *TP53 *locus. First, we assayed (in terms of sensitivity and specificity) the HRM technique, by comparing the results with the classic Sanger sequencing method, used here as the gold standard reference. Second, we propose solutions for genotyping challenges (discrimination of the 3 genotypes, simultaneous genotyping of 2 or more SNPs) that are sometimes encountered when using a classical HRM approach.

## Methods

### Origin of DNA samples

Mutation screening of the entire *TP53 *locus was performed on 47 DNA samples including lymphocyte DNA from 25 Li-Fraumeni patients, DNA from lymphoblastoid cell lines derived from 15 familial breast cancer patients, and DNA from 7 hemizygous (at the *TP53 *locus) breast tumor cell lines (Garritano et al, in preparation).

Genotyping of 30 SNPs located within the *TP53 *locus was performed on 270 DNA samples from the Coriell Repository, corresponding to 90 Caucasians, 90 East Asians, and 90 Africans.

This mutation screening and genotyping project received approval from the IARC Institutional Review Board and from the Brazilian center from which we received the Li-Fraumeni patient samples. It was conducted according to the Declaration of Helsinki Principles.

### Mutation screening/SNP discovery using HRM

PCR was performed in 8 μl reactions containing 20 ng of template DNA, 1.5 mM MgCl_2_, 265 μM dNTP, 400 nM forward and reverse primers, 0.8X LCGreen^® ^Plus (Idaho Technology, Salt Lake City, Utah, USA), 0.04 U/μl of Platinum^® ^Taq Polymerase, and 1× PCR buffer supplied by the manufacturer (Invitrogen, Paisley, Scotland).

The HRM process consists in performing the PCR in the presence of the DNA binding dye LC Green^®^, monitoring the progressive change in fluorescence caused by release of the dye from a DNA duplex as it is denatured by increasing the temperature, collecting a high resolution melting curve, and identifying the samples with melting curve aberrations indicative of the presence of a sequence variant. Fluorescence intensity as a function of temperature, monitored by the LightScanner^® ^instrument (Idaho Technology, Salt Lake City, Utah, USA), can reveal very small changes in the melting curve shape, when analyzed with the LightScanner^® ^software using the "Scanning" mode (Idaho Technology, Salt Lake City, Utah, USA).

### Genotyping using HRM

We designed pairs of primers flanking each SNP [See Additional file 1] to amplify DNA fragments shorter than 400 bp. In some instances, HRM can directly discriminate all 3 genotypes (common homozygotes, heterozygotes and rare homozygotes) of a polymorphism. However, for the majority of *TP53 *amplicons, genotyping using spike-in control DNA was performed to allow distinction of rare homozygotes from common homozygotes. In brief, genomic DNAs were mixed with an equal amount of DNA from a known major allele homozygous subject to allow formation of heteroduplexes. This strategy converts the minor allele homozygotes into heterozygotes, rendering them distinguishable from the major allele homozygous samples. The scoring of genotypes obtained with spike-in experiments was managed via automated procedures. For instance, we have developed a Laboratory Information Management Systems (LIMS) where results generated from a standard HRM genotyping plate and a corresponding spike-in genotyping plate are automatically converted into a final genotype call [28]. The program is also capable of rejecting samples that show unacceptable calls.

For amplicons containing two or more SNPs, sensitivity of mutation scanning may be decreased by producing complex melting curve data, and a different genotyping strategy had to be applied. This second strategy relies on an unlabelled probe-based genotyping analysis followed by mutation scanning, where the probe is designed to target the SNP(s) of interest. Both probe-amplicon duplex and whole amplicon duplex melting regions can be observed from the same melting run, in two distinct temperature windows, allowing genotyping and mutation scanning analyses to be performed simultaneously. Stratification of the samples according to their genotypes at the common variant positions prior to mutation scanning analysis reduces the noise and enhances the sensitivity for the detection of rare or unknown variants.

In practice, unlabeled 3' blocked probes targeting each common SNP were designed. PCR were performed in presence of a DNA dye (Here LC Green^®^) and oligonucleotides serving as probes were blocked at the 3' end to prevent extension during amplification. All genotyping assays were performed as a nested PCR, to ensure a good amplification of the region of interest. The primary PCR used standard conditions, whereas the secondary PCR included the unlabelled probe (500 nM) and was asymmetric so that more copies of the strand to which the probe anneals were produced. The ratio between the nested PCR primers was 1:5 (100 nM:500 nM) with an excess of the primer for the strand that is complementary to the probe. This favours probe-target annealing and reduces competition with the complementary strand [29]. Thus, this protocol produced sufficient double-stranded product for amplicon melting and enough single stranded product for probe annealing [30]. The analysis proceeds in two steps. The first step consists in analyzing the melting curve in the region corresponding to the probe T_m_. This step stratifies the samples into three groups based on the genotypes of the common SNP. The second analysis step consists in performing mutation scanning of the genotype-defined subgroups in the region corresponding to the amplicon T_m_, to identify the samples that are heterozygous for any rare sequence variants. For the amplicon containing SNPs rs9894946 (common) and rs17883532 (rare) the probe was designed to perfectly complement the rs9894946 T allele (GGAGCTCAGTAC**T**GCCTGCCC, the variable nucleotide is indicated in bold). For the amplicon containing SNPs rs858528, rs1641548, and rs1641549, two probes were designed. The first probe was designed to perfectly complement the rs858528 G allele (GCAGAGC**G**AGACTCAAAA). The second probe was designed to complement the rs1641548 G allele and rs1641549 A allele (TTAACC**G**GGC**A**TGATGGCAG, the variable nucleotides corresponding to SNPs rs1641548 and rs1641549 are indicated in bold). Probes were designed to have different T_m _(63°C and 54°C, respectively), in order not to interfere with each other in the melting data analysis.

## Results

### Mutation Scanning

During the course of a project to mutation screen the entire *TP53 *locus by direct resequencing from a set of 47 samples, we took delivery of a High Resolution Melt instrument. To assess the sensitivity and specificity of HRM for mutation scanning, we undertook mutation screening of the last 21 *TP53 *amplicons and of 1 amplicon corresponding to the proximal promoter region of the gene (from a total of 67 amplicons) by both full-sequence resequencing and HRM in a single pass experiment (Table 1).

**Table 1:** Oligonucleotide primer sequences used for comparison of HRM and sequencing sensitivity and specificity.

Amplicon	Forward sequence 5'>3'	Reverse sequence 5'>3'	Location	size
3	CGGGACGTGAAAGGTTAGAA	TTTTGGGGTGGAAAATTCTG	promoter	653
39	TGGCCATCTACAAGCAGTCA	ACACGCAAATTTCCTTCCAC	exon5-intron5	211
40	CATGAGCGCTGCTCAGATAG	CAGTTGCAAACCAGACCTCA	exon6	234
41	GTGGAAGGAAATTTGCGTGT	TTGCACATCTCATGGGGTTA	intron6	212
43	TGGCTCTGACTGTACCACCA	TCTACTCCCAACCACCCTTG	intron 7	371
44	CTGGAAGACTCCAGGTCAGG	AGCTGTTCCGTCCCAGTAGA	intron7	383
46	GCGCACAGAGGAAGAGAATC	TGAAAGCTGGTCTGGTCCTT	intron9	452
47	GCAGTGATGCCTCAAAGACA	GCAGGCTAGGCTAAGCTATGA	intron9	280
48	TGACTTTGCCTGATACAGATGC	TAGCTACTGGGGAGGCAGAG	intron9	596
49	GGCCTGCCTAGCCTACTTTT	GTAGCAGGCGCTTGTAGTCC	intron9	578
50B	GACTACAAGCGCCTGCTACC	TTTCATGCAACCATGCTGTT	intron9	614
51	CCCTACAGTTGGGCAAAGTC	CGACTGTGCCTCGTTTCTTT	intron9	491
52A	CCTGGGCGATAGAGTGAGAC	GGCTGGACTCAAACTCTTGG	intron9	134
52B	GTCGCATGCACATGTAGTCC	CTTGAGTTCCAAGGCCTCAT	intron9	635
53	ACTTCTCCCCCTCCTCTGTT	CCTGGGTTTGGATGTTCTGT	exon10-intron10	348
55	TATACTCAGCCCTGCCATGC	GGACTTCAGGTGGCTGTAGG	intron10	603
57	TTTGGGTCTTTGAACCCTTG	GTGGTTTCAAGGCCAGATGT	exon11 (3'UTR)	400
58	GGCCCACTTCACCGTACTAA	AAGCGAGACCCAGTCTCAAA	exon11 (3'UTR)	485
59	AAGGAAATCTCACCCCATCC	AAATGCAGATGTGCTTGCAG	exon11 (3'UTR)	456
60	TTGAGACTGGGTCTCGCTTT	CAGTCTCCAGCCTTTGTTCC		566
61	AAAACTTTGCTGCCACCTGT	ATCCTGCCACTTTCTGATGG		415
62	GCCTCTCACCAAGGATTACG	CCTGGACAGTAGCACCCACT		535

Nine of the amplicons were <400 bp in length, with an average length of 286 bp. For these, the sensitivity and specificity of HRM for sequence variant detection were 1.0 (38 true positive/(38 true positive + 0 false negative)), and 0.83, (295 true negative/(60 false positive + 295 true negative)), respectively.

Thirteen of the amplicons were >400 bp in length, with an average length of 544 bp. For these, the sensitivity and specificity of HRM for sequence variant detection were 0.81 (105 true positive/(105 true positive + 23 false negative)), and 0.84, (339 true negative/(69 false positive + 339 true negative)), respectively. Of note, the variant rs17551157, insertion of a cytosine following a 7 cytosine mononucleotide run in the TP53 promoter region, was undetectable in an amplicon of 653 bp.

The joint dropout rate from PCR, sequencing, and or HRM was 6.8%. Neither PCR-sequencing nor PCR-HRM had a single pass dropout rate exceeding 5%, thus staying above our general research mutation screening success rate target of 95%.

### Genotyping

We performed genotyping of 30 SNPs located within the *TP53 *locus on 270 DNA samples from the Coriell Repository.

**Figure 1:**
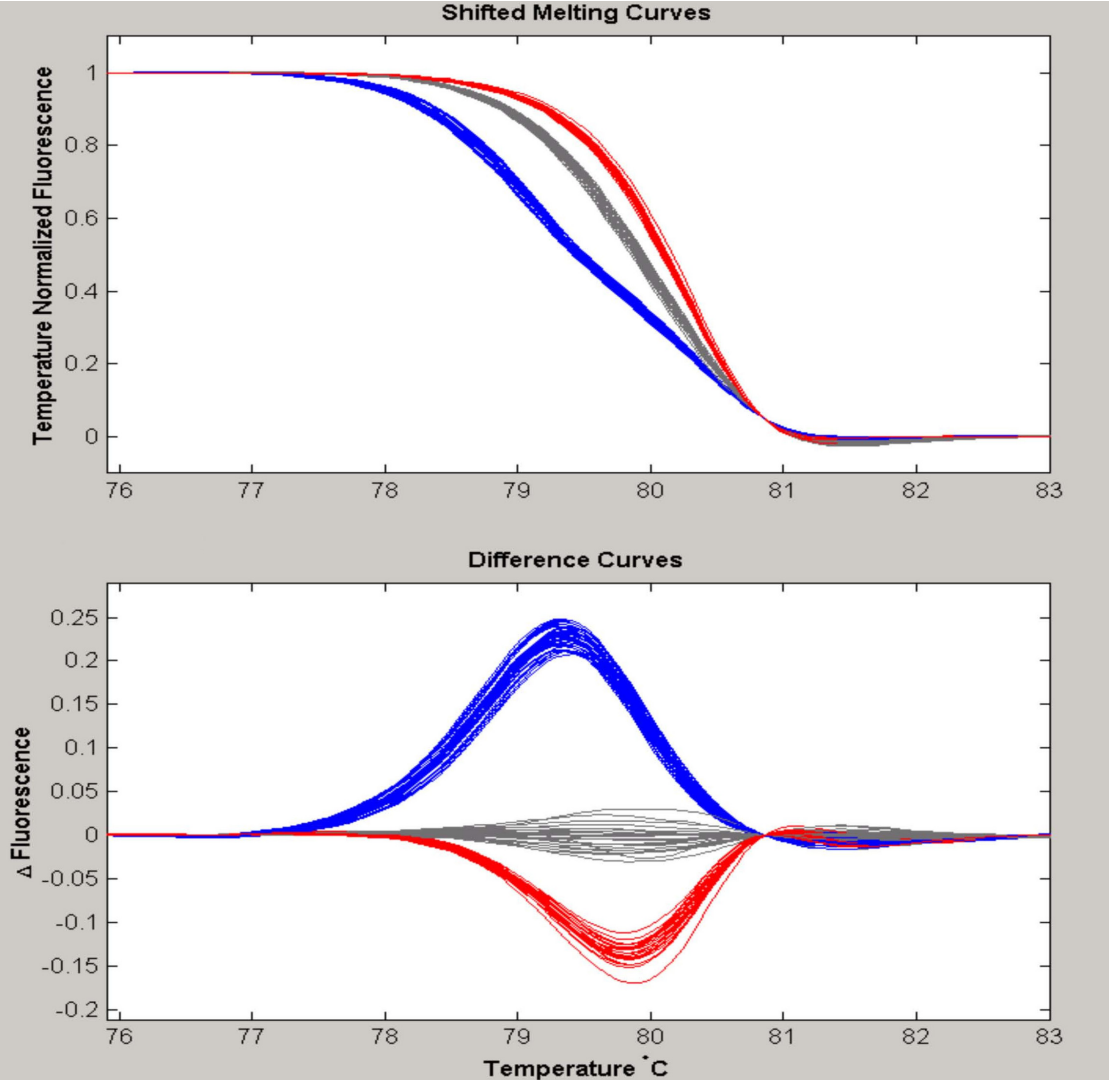
**Genotyping of SNP rs9903378**. The three groups are well distinguished: TT in grey, GG in red and TG in blue.

In some cases, it was possible to distinguish directly the three different genotypes of a SNP using standard HRM analysis of the amplicon of interest. Figure 1 displays the melting curve analysis of the SNP rs9903378, a T>G substitution. In this experiment, it was possible to discriminate the three groups corresponding to each genotype directly (common homozygotes TT, heterozygotes TG, and rare homozygotes GG) (Figure 1). This SNP resides in a T-rich sequence that has a low melting temperature. The GG samples have a melting curve different from the common homozygotes TT; evidently, the G interrupts the long poli-Ts and markedly increases the melting point of the amplicon.

**Figure 2:**
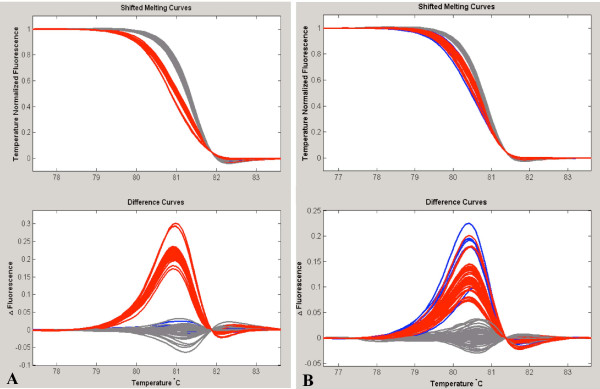
**Genotyping using spike-in control DNA to distinguish common homozygotes from rare homozygotes**. **A**. The melting curves of AG heterozygotes (in red) are distinguished from homozygous AA (in grey). Homozygous GG samples (in blue) are not distinguished from the common AA homozygous samples.**B **After spike-in of a homozygous AA sample, the GG samples are converted into AG heterozygotes (in blue), and they are now distinguished from AA samples.

**Figure 3:**
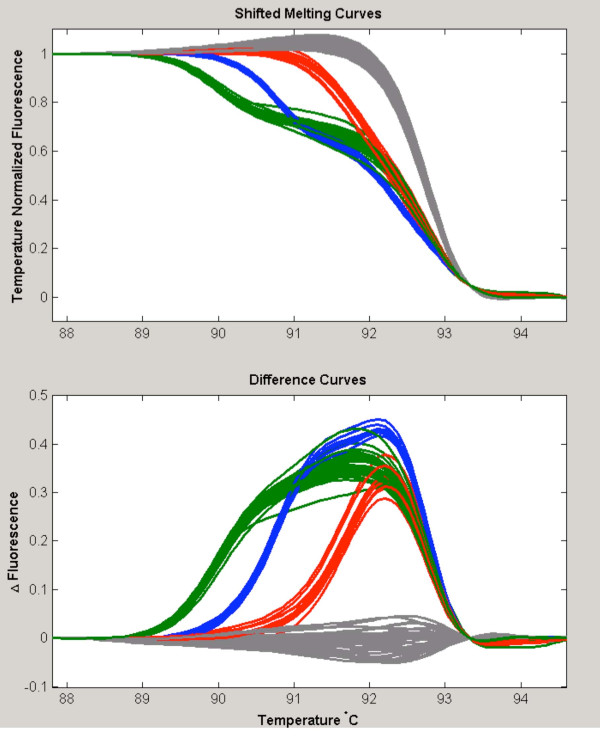
**Simultaneous genotyping of two or more SNPs within the same amplicon using the classic HRM approach**. There are two SNPs in this amplicon: rs17880560 and rs1614984. In grey, samples homozygous for both SNPs; in red, samples homozygous for rs17880560 (delCACGGC/delCACGGC) and heterozygous for rs1614984 (C/T). In blue, samples homozygous for rs1614984 (C/C) and heterozygous for rs17880560 (insCACGGC/delCACGGC). In green, samples heterozygous for both SNPs.

However, we found direct detection of minor allele homozygotes to be the exception rather than the rule, at least when using the mutation scanning approach for amplicons in the 200 bp to 400 bp length range. In this context, spike-in experiments provided an approach to detection of minor allele homozygotes. As an example, melting analyses for the SNP rs17881035 are displayed in Figure 2 (panels A, B). Applying standard melting curve analysis (Figure 2A), we observed that the heterozygous AG samples have a distinct melting curve profile compared to the common homozygote AA samples. However, the minor allele homozygote GG samples were not distinguished from the common homozygous AA samples because the difference between their T_m _was insufficient. In a second experiment (Figure 2B), each sample was mixed with an equal quantity of DNA from an AA homozygote (a pre-PCR spike-in experiment). This strategy in effect converts the minor allele GG homozygotes into GT heterozygotes, rendering them distinguishable from AA samples. In some instances, HRM can directly discriminate all of the genotypes of an amplicon that contains two SNPs. As an example, a 130 bp amplicon carrying SNPs rs17880560 and rs1614984 is displayed on Figure 3.

**Figure 4:**
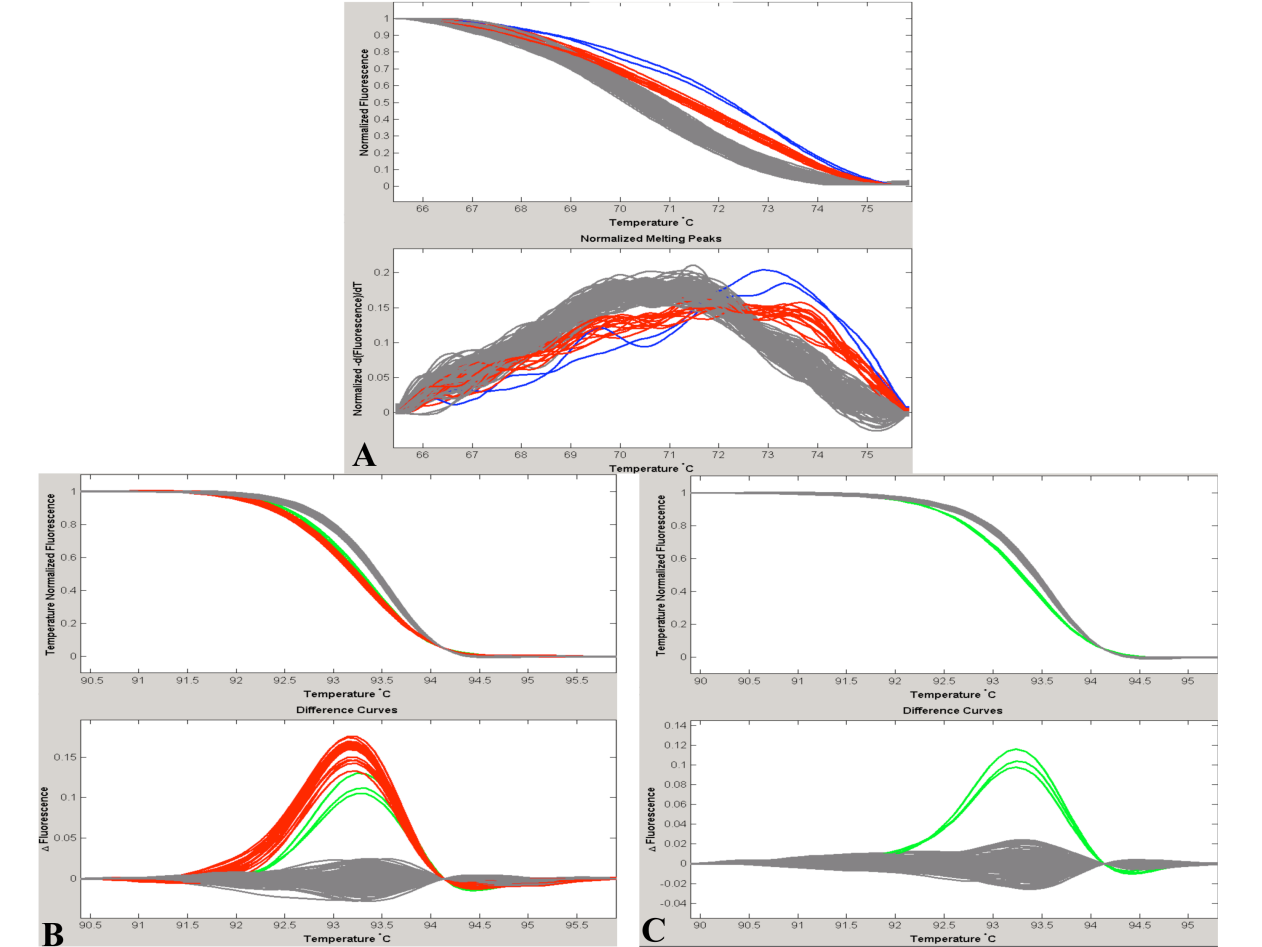
**Simultaneous genotyping of common SNP rs9894946 and rare SNP rs17883532**. **A**. In mutation scanning mode, heterozygous samples for either the first (in red) or the second SNP (in green) have virtually indistinguishable melting curves. **B**. In genotyping mode using an unlabeled probe for rs9894946, the 3 genotypes are distinguisable (CC in gray, CT in red, TT in blue). **C**. Mutation scanning of homozygous rs9894946 CC subset reveals heterozygous rs17883532 CT (In green).

Nevertheless, we have encountered examples where HRM cannot discriminate heterozygous samples for SNP1 from heterozygous samples for SNP2. In Figure 4 (panels A, B, C), we present an example and solution for an amplicon that contains two SNPs (rs9894946 and rs17883532) not directly distinguishable from each other. Heterozygous samples for either the first or the second SNP show almost indistinguishable melting curves (Figure 4A). We then designed an unlabeled 3' blocked probe that hybridizes to the region of sequence specific for the more common SNP rs9894946 [31]. Results are displayed in Figure 4 panels B, C. The first analysis step stratifies the samples into three groups based on the genotypes of rs9894946 (Figure 4B). In a second analysis step, mutation scanning of the genotype-defined subgroups in the region corresponding to the amplicon T_m _is performed, in order to identify the samples that are heterozygous for any other rare sequence variants. In this example, we found three heterozygous subjects for the SNP rs17883532 in 270 samples (Figure 4C). All these heterozygous subjects were homozygous for the major allele of SNP rs9894946. This solution is acceptable only if the second SNP is rare because the mutation scanning applied after the stratification of samples according the melting profile of the common SNP targeted by the unlabeled probe may not distinguish rare from common homozygous samples.

**Figure 5:**
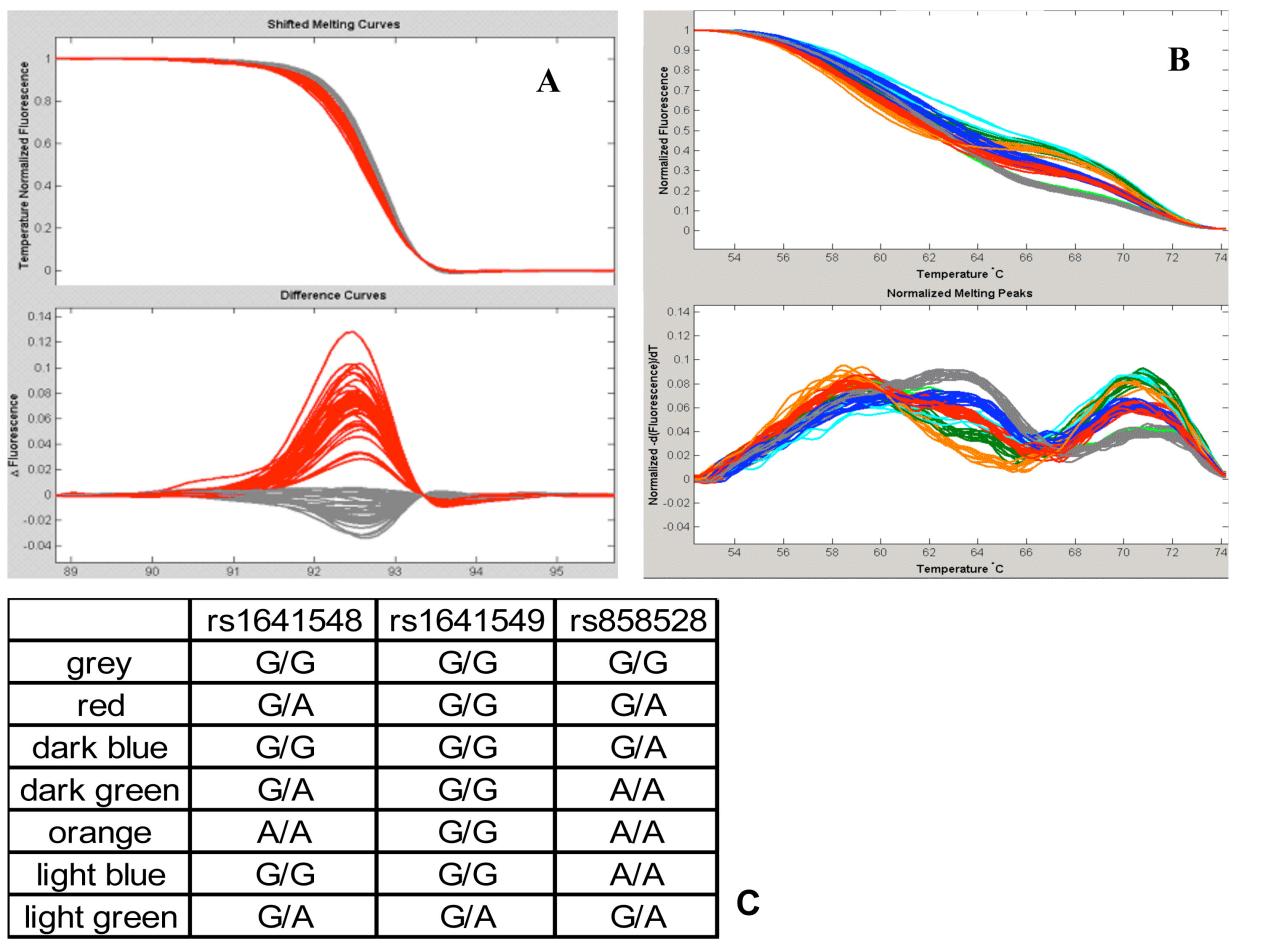
**Simultaneous genotyping of rs858528, rs1641548, and rs1641549 using two unlabeled probes**. **A **Samples that are heterozygous for rs858528, rs1641548, and rs1641549 have essentially indistinguishable melting curves (in red). **B **Genotyping using two unlabeled probes. Probe 1 targets the G allele of rs858528 and probe 2 targets the G allele of rs1641548 and the A allele of rs1641549. **C **Each distinct melting profile from panel B corresponds to a combination of genotypes of the three SNPs found in our population.

During the course of our TP53 study, we faced another particular situation, where two or more common SNPs lie in the same amplicon. In such a case, it may be necessary to use more than one unlabelled probe. For instance, one of our *TP53 *amplicons contains two SNPs: rs1641548 and rs1641549. These are only 4 bp apart, and both are A-to-G variants. Samples that are heterozygous for either the first or the second SNP have essentially indistinguishable melting curves (Figure 5A). Moreover, this amplicon also contains the SNP rs858528. In this case, two probes were designed. The first probe was designed to perfectly complement the rs858528 G allele. The second probe was designed to complement the rs1641548 G allele and rs1641549 A allele. Thus, homozygotes for the rs858528/rs1641548/rs1641549 haplotype G-G-A match both probes exactly and therefore have the highest T_m _across the compound-melting interval. Figure 5B and 5C show various genotypes combinations of the three SNPs found in our sample series.

**Figure 6:**
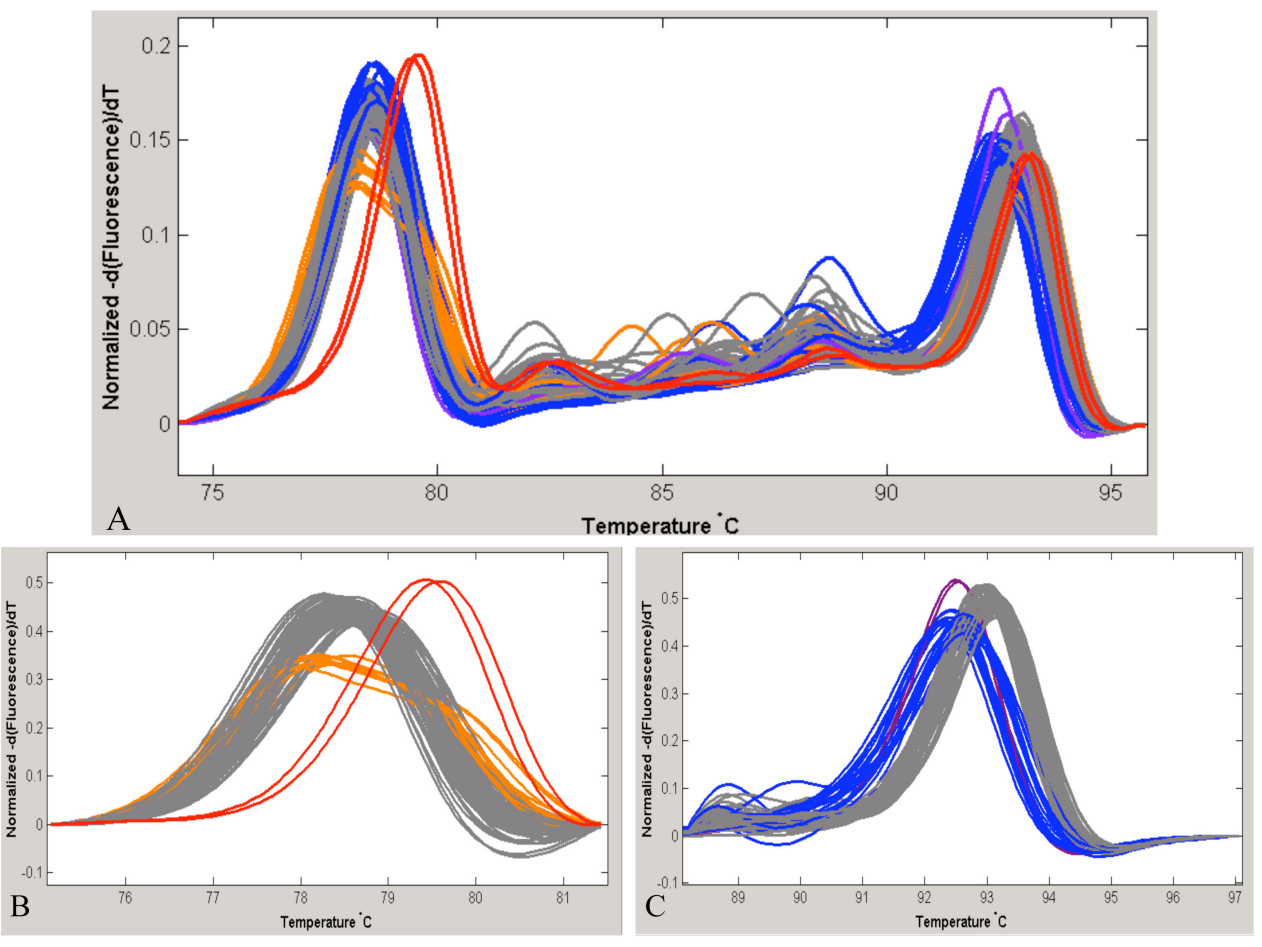
**An amplicon containing rs9903378 and an amplicon containing rs9894946 were amplified in a multiplex PCR**. **A **The melting curves of the different genotype combinations of the two SNPs show different profiles. In gray TT-CC, in blue TT-CT, in red GG-CC, in orange TG-CC and in purple TT-TT, respectively for rs9903378 and rs9894946. **B **The analysis was performed in the region of melting temperature of rs9903378 (75–80°C). in gray TT, in orange TG in red GG. **C **The analysis was performed in the region of melting temperature of rs9894946 (90–95°C). in gray CC, in blue CT in purple TT.

Finally, we evaluated whether the different genotypes from 2 independent PCR products could be discriminated from one single melting curve analysis. Since the amplicon containing SNP rs9903378 (which can be directly genotyped, see Figure 1) and the amplicon containing SNP rs9894946 (for which a specific probe had to be designed, see Figure 4) showed different T_m _(range 75–80°C and 90–95°C, respectively), they were selected to conduct the experiment. Both SNP containing fragments were amplified in a single PCR, and HRM analysis was conducted on the multiplex PCR product. In this last experiment, conditions of the multiplex PCR slightly differed from conditions of the simplex PCR, in order to achieve simultaneous amplification of both amplicons in a single reaction. In particular, the primer concentration for the amplicon containing the rs9903378 was decreased from 400 nM to 300 nM because at higher concentration only this amplicon was amplified (data not shown). Melting curves of the multiplex PCR products showed different patterns depending on the genotype combinations for the 2 SNPs (Figure 6). Using the "genotyping" mode of LightScanner^® ^software, it was possible to distinguish them from each other in a single melting curve analysis (Figure 6A). However, especially when analyzing a large number of samples (>80), resolution can be improved by performing the HRM analysis of the multiplex PCR products in two steps, that is by analyzing the 2 melting regions corresponding to the 2 DNA fragments separately (Figure 6B and 6C). Thus, our results demonstrate that HRM genotyping of multiplex PCR products is feasible and cost and time effective.

## Discussion

In this manuscript, we assessed the sensitivity and specificity of HRM for mutation screening by comparing it head to head with the direct resequencing of 21 *TP53 *locus amplicons on 47 DNA samples. A second application of the HRM analysis was the genotyping of 30 known SNPs within this gene on 270 DNA samples.

In mutation scanning mode, the sensitivity and specificity of HRM were 1.0 and 0.80, respectively, for amplicons of <400 bp, and 0.81 and 0.84, respectively, for amplicons of >400 bp.

Recent studies have validated HRM for screening of number genes of clinical significance [21-26]. These studies also reported a sensitivity of HRM close to 100%, except in the situation were amplicons have a high GC content [26]. We also encountered a similar situation with one *TP53 *GC-rich amplicon (see below). However, in the previous studies, the HRM technique was evaluated only on partial or full coding sequence(s) of the genes of interest. For instance, more than 80% of *TP53 *mutation studies focus on exons 5–8 (residues 126–306) because most mutations are localized in the DNA binding domain of the protein (residues 100–300) [21,22]. However, in one study where the HRM analysis was extended to the entire coding exon of *TP53*, 41% of the alterations fall outside exons 5–8 [22]. Thus partial scanning of *TP53 *sequence may lead to a bias in the mutation analysis. Following this idea, we aimed to evaluate a set of HRM assays that sample the entire *TP53 *locus, including one or more amplicons from the proximal promoter, coding exons, introns, and 3' UTR. Our study thereby provides a broader view of the strengths and limitations of HRM-based techniques. However, the *TP53 *amplicons used in the present study were not designed specifically for HRM but rather for mutation screening of the entire *TP53 *locus by direct resequencing in the context of a Li-Fraumeni syndrome-related study (Garritano *et al*, in preparation). Consequently, some of the amplicons were longer than the optimum for HRM mutation scanning. Nonetheless, we have shown that the sensitivity of HRM for mutation screening is very high, especially for amplicons <400 bp.

For genotyping applications, especially for intronic SNPs, primers were sometimes designed quite far from the SNP of interest to avoid unspecific amplification. Despite the length of the amplicons used, we obtained full concordances between HRM genotyping calls and results of direct sequencing. Moreover, using an amplicon of >400 bp, we succeeded to simultaneously genotype two SNPs located approximately 200 bp apart from each other (SNP rs858528 and rs1641549), thus reducing the number of PCR reactions and improving time and cost effectiveness.

In mutation scanning mode, HRM tended to call as "variant" some DNA samples that actually were wild type. To minimize the frequency of false positive "variant" calls, it is recommended to standardize DNA preparation, storage methods, and storage conditions. Because the sensitivity and specificity of HRM are exquisitely dependent on the melting temperature of each individual sample, variation in salt or buffer concentration carried into PCR reactions along with substrate DNA can generate heterogeneous melting profiles. If needed, to reduce sample-to-sample heterogeneity, it can also be useful to perform a nested PCR and the HRM assay on the secondary PCR.

In the course of our work, we have observed one potential weakness in HRM: the technique may have limited sensitivity for single nucleotide insertion-deletion variants located immediately adjacent to mononucleotide runs of sufficient length that they stutter during PCR. In this work, SNP rs17551157, an insertion of a cytosine adjacent to a 7 cytosine repeat within TP53 proximal promoter, was undetectable in a 653 bp HRM amplicon. A similar situation was also encountered during a large-scale case/control mutation scanning of *ATM *performed in our laboratory, where insertion of an adenosine adjacent to an intronic run of 10 thymines (rs3218681) was also undetectable by HRM mutation scanning. In both cases, the sequencing chromatograms revealed PCR stuttering at the mononucleotide run (data not shown). Such mononucleotide runs are relatively uncommon within the ORF of protein coding genes. Nonetheless, we suggest that at the outset of an HRM based mutation-screening project, investigators check the ORF of the gene of interest for mononucleotide runs that could create such a problem. If any are present, apply a different mutation screening technique to the relevant amplicon.

From our extensive mutation screening of the *TP53 *locus, we found that HRM provides sensitive assays both for detection of new sequence variants and genotyping of known polymorphisms. Table 2 summarizes the various HRM approaches for the different genetic contexts that we have considered, according to the number of SNPs present in each amplicon and to their frequencies in the studied population. In our experience, selecting an appropriate HRM analysis strategy depends both on study size and the number of known common polymorphisms in a given amplicon. For relatively small mutation screening studies, it may be reasonable to sequence all samples that appear to contain a sequence variant. In this case, amplicons known (or found) to contain a common variant can be PCR amplified in duplicate, once as a standard analysis and once as a spike-in analysis. The former will detect the presence of heterozygous variants and the latter will detect the presence of minor allele homozygotes. All samples with HRM curves that differ from the major allele homozygotes curves would then be queued for sequencing.

**Table 2:** Summary of different approaches utilized for each genetic situations.

**Genetic situation**	**HRM solutions**
No common SNP in the amplicon	Direct HRM analysis

Only one common SNP in the amplicon	Sometimes it is possible to distinguish directly the three groups (Figure 1) Otherwise, spike-in or unlabelled probe is needed (Figure 2)

Two SNPs in the amplicon, one is common the other is rare	Spike-in when possible (Figure 3); otherwise, use unlabeled probe for the common SNP, and perform mutation scanning for the rare one (Figure 4)

Two or more common SNPs in the amplicon	Use an unlabeled probe for each SNP (Figure 5)
Two SNPs in two different amplicons	When T_m _of 2 amplicons is different, it is possible to perform multiplex PCR (Figure 6)

In large-scale mutation screening studies, there may be cost benefit to enabling HRM determination of common SNP genotypes prior to mutation scanning, so that only samples showing a variant HRM curve not attributable to the presence of a common SNP are queued for sequencing.

If an amplicon contains a common variant, this variant can mask the presence of a rare variant that might have the same melting profile. Without a discrimination step, one has to either 1) sequence all the heterozygous samples, even though most will be due to a common SNP or 2) accept failure to detect rare variants that have the same melting profile as the common SNP. Inclusion of a discrimination step, which can be achieved with little added cost and with no extra PCR reactions, allows assigning the common SNP by genotyping and simultaneously queuing the rare variant heterozygotes for identification by sequencing.

## Conclusion

HRM is a simple and cost effective post-PCR technique that can be used for high-throughput mutation scanning and genotyping in a small laboratory environment. It is inexpensive, flexible, and only mildly constrained by primer design. HRM reactions are closed-tube, which reduces risk of contamination. In addition, HRM assays are non-destructive so that the actual sample used in mutation scanning can serve as a sequencing template.

## Abbreviations

dHPLC: denaturated High-Performance Liquid Chromatography; HRM: High-Resolution Melting curve analysis; SNP: Single Nucleotide Polymorphisms.

## Authors' contributions

SG carried out the experiments and drafted the manuscript; FG participated in its coordination and helped to draft the manuscript; CV developed the Laboratory Information Management Systems (LIMS); TND participated in design of the unlabeled 3' blocked probe assays for each common SNP; FLK participated in the development of the laboratory workflow; DD participated in the experiment design; FL participated in its coordination and helped to draft the manuscript; SL participated in its coordination and helped to draft the manuscript; SVT conceived the study, participated in its design and coordination and helped to draft the manuscript. All authors read and approved the final manuscript.

## Supplementary Material

Additional file 1 **Primers sequences for *TP53 *SNPs analyzed in the present study.** The data provided correspond to the oligonucleotide sequences used to analyze *TP53 *SNPs. Click here for file
