# Supplementary material for: Determining the effectiveness of High Resolution Melting analysis for SNP genotyping and mutation scanning at the TP53 locus
Source: BMC Genet. 2009 Feb 17;10:5. doi: 10.1186/1471-2156-10-5 (PMC2648999; doi:10.1186/1471-2156-10-5)
Supplement: Additional file 1 — Primers sequences for TP53 SNPs analyzed in the present study. The data provided correspond to the oligonucleotide sequences used to analyze TP53 SNPs. [file 1471-2156-10-5-S1.doc]

**Additional file 1.** Primers sequences for *TP53* SNPs analyzed in the present study

| SNPs | Forward sequence 5’ > 3’ | Reverse sequence 5’ > 3’ | Amplicon size (bp) |
| --- | --- | --- | --- |
| rs2287499 | ACTCTGTTTCCAGGGGAGTG | GGTTGTCCCCAGATCCTGT | 93 |
| rs17551157 | CTACGCTCCCCCTACCGAGT | AAGAGGTGCAAGACCTGCTG | 94 |
| rs17883353 | GGGCGACAAGAACGAAAC | ACAACATGAACGAATGTCAGA | 113 |
| rs17882227 | TGGCCTTTTGAGTTGTTTCC | CGCAATTCCCCTCCTAAGTA | 125 |
| rs17885845 | GGAATTGCGAGTTTGGAAGT | CTAATGTCCGGAAGGCTGTG | 146 |
| rs9903378 | TTCAGTATTTGCCTTGTCCTGTT | CCATAAAGGTCCTAAAAGAAAATG | 100 |
| rs17881035 | TTAACCCCAGGGTCATGAAG | TGACTCCTACACCTCACACCA | 135 |
| rs11656607 | GCAGGGGATCATTTGAGG | TGGCTTCGAAAACAAGTTGG | 290 |
| rs5819163 | ACATTACCCCCATACAATGA | GTGGCTGCTGGTATCAGTCTT | 120 |
| rs2078486 | AGTGGGGGTGGGAGCAGTA | TGCAATTGTTCTATTTCACTTGTTC | 94 |
| rs1642782 | AAGATGCTGGTCCACACAGG | ACCTGCTTGGGCTCAATAAA | 104 |
| rs12944939 | GTTAACAGGAGGTGGGAGCA | AGAGACAGGGCTTTGCATGT | 132 |
| rs8079544 | CAGCCATTCTTTTCCTGCTC | CTTCCAACCCTGGGTCAC | 108 |
| rs1642785 | CCCCTCTGAGTCAGGAAACA | TCCCACAGGTCTCTGCTAGG | 114 |
| rs17878362, rs17883323 | TTCCTGAAAACAACGTTCTGG | GGGGACTGTAGATGGGTGAA | 134 |
| rs1042522 | GAAGACCCAGGTCCAGATGA | TGGTAGGTTTTCTGGGAAGG | 146 |
| rs1794287 | TCCCAGCACTCTCAAAGAGG | CTCCATCTCCTGGCCTCA | 194 |
| rs1625895 | GGGTTAAGGGTGGTTGTCAG | TTGCACATCTCATGGGGTTA | 97 |
| rs12947788 | CTGGAAGACTCCAGGTCAGG | TGATGAGAGGTGGATGGGTAG | 135 |
| rs12949655 | CCAGGCTGGTCTCTAGCCTAC | TAGCAGGCGCTTGTAGTCCT | 357 |
| rs858528, rs1641548, rs1641549 | CAGTCGAGCCAGGCACAG | TGTGCCTCGTTTCTTTTCTTT | 359 |
| rs6503048 | TAATCCCAGCTACCTACTCG | CAATGCTTTGAAGGGCCTAA | 282 |
| rs17880560, rs1614984 | GCTCCATTCATAACTCAGGA | CCGTAATCCTTGGTGAGAGG | 130 |
| rs9894946, rs17883532 | TCAAAGGGCTTCTTGGGATA | AAGAGAGCAGTGGGTGATGG | 138 |
| rs17886760 | ATCACCCACTGCTCTCTTGG | CACCCACTGGACCCTAACAC | 104 |
